# Supplementary material for: The regulatory landscape of early maize inflorescence development
Source: Genome Biol. 2020 Jul 6;21:165. doi: 10.1186/s13059-020-02070-8 (PMC7336428; doi:10.1186/s13059-020-02070-8)
Supplement: Supplementary file 10 — Additional file 17. Review history. [file 13059_2020_2070_MOESM17_ESM.docx]

Review History

**First round of review**

**Reviewer 1**

**Are you able to assess all statistics in the manuscript, including the appropriateness of statistical tests used?**

Yes, and I have assessed the statistics in my report

**Comments to author:**

Eveland and colleagues have performed a detailed analysis of chromatin accessibility in developing maize inflorescence tissues.  There are a number of interesting observations in the manuscript.  This dataset is a valuable resource that could be used by other groups and also provides some new biological insights.  However, the presentation could be substantially improved, both for clarity and for succinctness.  In many places of the manuscript it was actually quite difficult to find truly new insights or impactful discoveries.  Instead the manuscript often became lists of differentially expressed genes or GO terms that didn't really add knowledge. I provide a series of comments/questions/suggestions that are meant to help the authors improve the communication of their work.  In many instances this paper would likely have been substantially improved if the authors had simply forced brevity.  The inclusion of extra cut-offs or analyses often made the work confusing or less powerful.

1. Use of v3.  The authors are using a relatively old version of the reference genome.  There may be a specific reason for this.  However, the use of v3 will really limit the utility of the results for other researchers.  Most recent work on putative enhancers or accessible regions of the genome are on B73v4 and many researchers will now by using v5.  I would strongly encourage the authors to move this work to v5 (or 4) and simply roll coordinates back to v3 if they have specific datasets or resources for that version they wish to incorporate.  

2.  Calling of peaks.  The paper is often difficult to follow when discussing accessible regions.  This is due to complications od shifting between different analyses methods and different filters.  While I can understand the uses of both of these I think it would significantly improve the manuscript to very early select a single cut-off (likely bc3) and just use this set throughout.  In addition, specific titles should be given to the regions called using the different analyses (short fragments, etc).

3.  GO analyses and specific examples.  The manuscript too often includes GO enrichments or specific genes as examples.  In many cases these do not provide strong support for specific findings of this work and simply increase the manuscript length.

Specific comments:
Lines 136-138; I am not really a big fan of acronyms but they might be useful here.  It would be nice to have specific terms for each kind of mapping/region that was called.  Later in the manuscript it often became difficult to follow which of these types of regions were being used.

Lines 141-150: There is too much detail here.  Pick a cut-off and justify why it captures biologically meaningful data.  In addition, whatever cut-off is used should be retained throughout the work.  Currently the shifting bc cut-offs make it seem like the authors were p-hacking to find significant results.

Line 174 - this bar plot is not as useful as a metaprofile would be to visualize the relative location of peaks relative to genes

Line 182: 'intergenic' a confusing term here as all things in this plot are intergenic.  Perhaps 'genomic distribution' or something else would be better.  It took me a bit to understand this plot.

Line 193: What are you doing with non-expressed genes?  Are they in the low expression group or are they excluded?

Figure 2: The colors used here are difficult to separate.  I would encourage the authors to use easily distinguished colors for the panels a and b

Lines 208-214:  This is a really interesting and important question.  How well does the variation in accessibility associate with dynamic gene expression.  I had a really tough time understanding the authors take-home point about this.  To me it looks like the correlation is quite poor.  Only a subset of regions with alter accessibility show altered expression and the directionality is not well correlated.  Are these associations statistically significant?  What is the proportion of positive and negative correlations?  The plot really doesn't show this well.  

Line 215-229: This is not well justified.  What are the authors trying to address here?  They are focused on the subset of genes with altered accessibility and expression.  Why are these more important or meaningful than if they looked at all genes with differential expression (but not necessarily altered accessibility)?  As expected they find some TFs with known functions.  The detailed analysis of TCP genes really doesn't seem to have a true take-home.  Instead this paragraph seems to provide a random set of observations that are not really testing specific concepts.  If the authors could show the TFs with altered accessibility and expression are more interesting or meaningful than TFs that only change in expression that could be intriguing.

Lines 230-247: Again - what is the goal here.  Why are these genes better than simply looking at DE genes?  These discussions of GO terms really don't seem to actually provide any insights.

Lines 253-255; This is a really nice result and highlights the value of these HS sites in finding potential TF binding sites.  

Lines 258-260: I am not convinced this observation is very useful.  Why is it important to note a slight difference in binding site size?  Is this statistically significant?

Lines 260-264: Go terms provide no value here.

Lines 273-288: This is an interesting analysis.  Using the accessible regions around FEA4 sites the authors find other TF motifs to suggest potential co-regulation.  What proportion of the FEA4 accessible regions contain this motif?  If you divide the genes near these peaks into those with and without and ABI3 motif due these two groups of genes show similar or distinct expression patterns in your samples?

Lines 293-296: Why use a higher (bc4) cut-off here?  What about KN1 binding sites?
Lines 297-307: I don't understand why groups of genes with positive and negative associations are grouped together here.  It seems you'd have more power to focus solely on the groups of genes for which there is a positive association or a negative association separately.

Lines 313 and on: Why are these enhancers?  Aren't they as likely to be distal repressors as enhancers?

Lines 318-320: The evidence for lncRNA overlap with distal regulators in plants is relatively sparse.  Many distal enhancers have little or no lncRNA detected.

Lines 322-380: This analysis really doesn't seem to fit well within this paper.  The authors identify putative lncRNAs.  It is not at all clear why they did not use previous lncRNAs that included these tissues and many others.  The observations about lncRNA distribution and location are very similar to previously published works.  Similarly, the analysis of chromatin near lncRNAs has been documented in several plants including maize.  This whole section really does not really associate these lncRNAs to the accessible regions the authors are studying or compare lncRNAs from accessible regions to those from inaccessible regions.  Unless this is better justified within this work it seems this should be moved to a separate work.

Lines 383-410: This is not a novel finding.  The Stam and Jiang groups nicely demonstrated that many accessible regions are located within annotated transposons.  The results in this work do not provide additional information beyond these earlier works and those are not cited here.

Lines 412-455:  This was an interesting analysis.  There are many issues with GWAS implementations and the idea here to focus on accessible regions to improve detection was interesting.  Once again, the authors switch to a different cutoff (bc2).

Lines 439-455: The authors really do not provide evidence for improvement of outcomes using their approach.  The overlap is quite limited (9 in common and 46/63 specific to genome wide or accessible region SNPs).  As expected some of the best candidates do overlap.  The authors find some cases in which the same gene was identified as significant due to different SNPs.  How often did this happen?  The authors describe an example of this but don't specify the frequency that this happens and can't resolve which SNP is more likely causative.  Finally the authors mention several candidates found using the MNase SNPs but not the genome wide examples.  This is cherry picking of some examples.  This really isn't a good quantitative estimate of the value of these approaches.

**Reviewer 2**

**Are you able to assess all statistics in the manuscript, including the appropriateness of statistical tests used?**

Yes, and I have assessed the statistics in my report

**Comments to author:**

The study by Parvathaneni, Bertolini et al using differential nuclease sensitivity mapping to reveal the regulatory landscape of developing maize inflorescences. Chromatin accessibility was identified in a manner that enabled footprint resolution providing data necessary for the construction of some key transcriptional modules that are important to the development of maize ear/tassels. The regions of differential nuclease sensitivity were highly concordant with in vivo ChIPseq data of key transcription factors (KN1/FEA4) supporting the high quality nature of the accessible chromatin regions identified. Additionally, a class of inflorescence enriched lncRNAs were also captured in this study. They were annotated and possible regulatory roles were explored. Lastly, focusing on traits associated with the tissues examined the intersection of trait associated SNPs from previously published GWAS and the newly identified accessible regions were evaluated. This analysis revealed a significant enrichment of trait associated SNPs in differential nuclease sensitivity sites.

Overall, the use of differential nuclease sensitivity mapping to identify the cis-regulatory landscape of the development of maize inflorescence tissues represents a valuable contribution to the field. This study is timely and it incorporates tissue specific DNA footprint analysis that enables the exploration of transcriptional factor regulatory pathways important to development of these tissues. The comments below are intended to improve this already strong manuscript.

Major comments:

1.     Footprint analysis using DNase I and/or Tn5 has revealed that some sites are sensitive to the biases associated with the enzyme being used. One way to identify these sites is to use a genomic DNA control and to perform footprint analysis on the regions found from using chromatin as the input material. Is this a concern when using MNase and if so, what is the most appropriate control to manage enzymatic bias in nuclease activity that plagues DNase-seq and ATAC-seq.
2.     It is stated there is a high rate of false negatives from the peak identification of nuclease sensitive sites. What is the evidence that adding more reads solves this solution, as replicates were combined for most additional analysis. Can individual replicates be used for analysis and figure creation (such as Figure 2b and 5c) instead of combining them (with the exception of footprinting where deep data are required)?
3.     Line 183. What is the biological rationale for why ear and tassel peak distance would be so different? Is this somehow related to technical quality of the data instead of biology?
4.     I'm confused why mid points were used for identification of DNA footprints. Why can't the first base be used to indicate regions that are sensitive to nuclease activity. Then footprints should emerge from regions of accessibility that have reduce or no nuclease activity, although they are surrounded by lots of nuclease activity.
5.     Please report how many intergenic lncRNAs were removed from the final list due to protein-coding capacity. This is especially important given the known issues with the annotation of the maize genome.
6.     Line 346. Around 30-40% of the enhancers identified from reference 16 are enriched for unannotated genes and lncRNAs. Therefore they are not classic enhancers.

Minor comments:

1.     Most of the opening of the results section could be moved to the methods section.
2.     Change "linc" to lnc

**Reviewer 3**

**Are you able to assess all statistics in the manuscript, including the appropriateness of statistical tests used?**

Yes, and I have assessed the statistics in my report

**Comments to author:**

Dear Authors,
The manuscript "The regulatory landscape of early maize inflorescence development" was a pleasure to read. Very important work, outstanding representation in the figures and an amazing wealth of data to dive in and continue the quest to understand and manipulate the switch between the female and male flowers in maize.
This paper is massive also in its current form and I needed several breaks to fully digest the content while reviewing. I could not find any concerns with the work or methods.
My only minor concern is the length and the possibility that valuable information might not be fully utilized by our research community simply because it is "hidden". The presented Discussion is a review paper in itself, and the Results sections could be split in two strong research papers. Just saying...

Great job and I will continue reading it over and over again to make sure not to miss any important information.

**Authors Response**

**Point-by-point responses to the reviewers’ comments:**

Reviewer 1:
Eveland and colleagues have performed a detailed analysis of chromatin accessibility in developing maize inflorescence tissues. There are a number of interesting observations in the manuscript. This dataset is a valuable resource that could be used by other groups and also provides some new biological insights. However, the presentation could be substantially improved, both for clarity and for succinctness. In many places of the manuscript it was actually quite difficult to find truly new insights or impactful discoveries. Instead the manuscript often became lists of differentially expressed genes or GO terms that didn't really add knowledge. I provide a series of comments/questions/suggestions that are meant to help the authors improve the communication of their work. In many instances this paper would likely have been substantially improved if the authors had simply forced brevity. The inclusion of extra cut-offs or analyses often made the work confusing or less powerful.

*Response: We appreciate Reviewer 1’s comments on improving the presentation of the manuscript for clarity and forcing brevity. We completely agree and have refined the technical detail, including the discussions of extra biological cut-offs. More detail is provided in individual comments below.*

1 – Use of v3. The authors are using a relatively old version of the reference genome. There may be a specific reason for this. However, the use of v3 will really limit the utility of the results for other researchers. Most recent work on putative enhancers or accessible regions of the genome are on B73v4 and many researchers will now by using v5. I would strongly encourage the authors to move this work to v5 (or 4).

*Response: We appreciate the reviewer’s concern with respect to comparing across datasets on a single reference genome. The issue we face is that the genome will continue to improve, requiring remapping of data. We have continued to use version 3 of the maize genome since we find the most recent gene model set from v3 to be most consistent with our experimental data in several instances compared to that of version 4. Other researchers in the community have also continued to work with v3 throughout the ‘v4 era’ and there have been several high-quality maize papers, particularly related to developmental genetics and genomics, published within the last six months or so that used the v3 genome, e.g., Knauer et al., Genome Res., 2019; Leiboff and Hake, Current Biol., 2019; Dong et al., Nat. Comm., 2019, Tian et al., Science, 2019. The popular DOE-backed plant comparative genomics database, Phytozome, continues to display v3 as the default maize genome.

Indeed, there are comparable datasets profiling regulatory elements in maize that have recently emerged (e.g., Ricci et al., 2019; Lu et al., 2019) and are displayed on v4. However, these and other datasets will soon need to be remapped to the recently released v5, yet the insights from these papers will not be lost. We do not feel our work is any less impactful having been mapped and analyzed based on v3, than it would be on v4. This manuscript was submitted before the release of v5. Although the v5 genome sequence is currently available, the gene models are being released in two batches and only the first has been released (other due upon its publication). We believe genetic and molecular insights from our analyses performed consistently in v3 with a solid set of gene models will not change much with reanalysis in another genome.

However, we do want our data to be readily accessible to the community for visualization and integrated analyses with the v5 maize genome, so we also mapped the MNase data to maize RefGen_v5 and called accessible regions using iSeg. These data, in addition to all of the processed data for v3, are available for download on GEO: Additional File 5: Dataset S4. We compared our mapping and peak calling results of the MNase-seq data between the two genomes and as expected, the were highly comparable. Mapping was 3-4% higher for v5, likely due to gap filling and additional scaffold assembly. Overlap of accessibility (bp match) between genomes was greater than 90% for both tassel and ear. Manual inspection on a genome browser indicated very high concordance in position of accessible regions between genomes in and around loci discussed in the manuscript. We have also been in discussion with MaizeGDB to host our v5 MNase-seq coordinates upon data release.*

2 – Calling of peaks. The paper is often difficult to follow when discussing accessible regions. This is due to complications of shifting between different analyses methods and different filters. While I can understand the uses of both of these, I think it would significantly improve the manuscript to select a common cut-off and use throughout. In addition, specific titles should be given to the regions called using the different analyses.

*Response: We completely agree with all of the points the reviewer makes here. We struggled with deciding whether to include the detailed information, which could be useful, but rather seems to cause confusion. We condensed the text on peak calling and biological cut-offs originally found in the beginning of the Results section, and moved it to the Methods section (now at lines 632-644).
We absolutely agree that terms should be designated for the different MNase-seq analyses and used throughout. We continue to call the Differential Nucleosome Sensitivity analysis DNS, but also now refer to the light small fragments as Light Coverage Small (LCS). This is first introduced in line 135: “.. small fragments (<131 bp) coverage from the light digests only, hereafter referred to as Light digest Coverage Small fragments (LCS)”, in the legend for Figure 1, and then used throughout the manuscript.*

3 – GO analyses and specific examples. The manuscript too often includes GO enrichments or specific genes as examples. In many cases, these do not provide strong support for specific findings of this work and simply increase the manuscript length.

*Response: We respect the reviewer’s opinion on this, but we disagree in most cases. We believe that the functional analyses of gene sets in contexts defined in the manuscript are highly relevant to the biological question, which laid the foundation for this work – to interrogate the regulatory space during early tassel and ear development, and to identify candidate regulatory loci that may underlie differences in their morphology or other aspects of biological relevance. Our desire was not only to provide a novel context-specific genomics resource, but to mine it for biological insights that can lead to testable hypotheses.

There are a number of places where we use Gene Ontology to try and capture hidden biological relevance. In particular, our discussion of genes associated with tassel- or ear-specific regulation is central to identifying factors that may underlie meristem fate more broadly (indeterminate tassel branches vs. determinate spikelets) but also that may be important targets for genome engineering in this important agronomic system. This is highlighted in the first part of the Discussion (lines 427-458) and we expect that discussion of biological insight coming from both of these areas will draw a wider reader base. We believe there are many readers who appreciate descriptive discussions of biology (including making inferences on candidate genes and functional enrichments) in the context of large multi-omics studies. It also facilitates gene curation and enhances manuscript citations. A grand challenge in biology currently is characterizing the function of every gene in the genome. In maize and other crops we have an understanding of what only a small fraction of the genes do, e.g., for many transcriptional regulators function is inferred based on a single family member for which there is data available. Enhancing our understanding of gene function will require reporting on previously uncharacterized genes in various contexts so that curation can be improved through literature searches.
In a 2014 manuscript by corresponding author Eveland (Eveland et al., in Genome Res. 2014), a similar approach was taken to present gene lists and GO terms associated with certain meristem pathways in maize. There have been documentable cases of where researchers from various labs followed up on genes in those lists. We believe these curated gene lists and functional analyses are valuable for these reasons.

We do agree, however, that there are several places in the manuscript where biological insight is discussed that indeed could be condensed so to keep the interest of readers while also highlighting the most important biology. We condensed some of these areas, including removal of extended discussion on TFs up-regulated in tassel, one of the GO analyses that we agreed was not as useful (genes bound by KN1 and FEA4; commented on below), an entire section in the discussion around ABI3 related genes and potential interactions with FEA4, and condensed part of the discussion on TCP TFs. (more details in responses below)*

Specific comments:

Lines 136-138; I am not really a big fan of acronyms but they might be useful here. It would be nice to have specific terms for each kind of mapping/region that was called. Later in the manuscript it often became difficult to follow which of these types of regions were being used.

*Response: We really appreciate this suggestion and also feel this will help the reader navigate through the different analyses. The changes we made are listed in response to comment #2 above.*

Lines 141-150: There is too much detail here. Pick a cut-off and justify why it captures biologically meaningful data. In addition, whatever cut-off is used should be retained throughout the work. Currently the shifting bc cut-offs make it seem like the authors were p-hacking to find significant results.

*Response: We completely agree that we originally provided too much detail here, which can certainly cause confusion rather than clarity. As also suggested by reviewer 2, much of this has been condensed and/or moved to the methods section (now lines 632-644).

There is rationale for using different cut-offs in the peak calling algorithm to address specific biological questions, i.e., selected to closely match biologically meaningful genome coverage ranges. Meaningful guidelines for our peak calling stringency come from the analysis of MNase sensitivity as measured using the same data type (DNS-seq) and species (maize); Rodgers-Melnick et al., PNAS, 2016. In the previous version of the manuscript, we did compare the different cut-offs based on certain criteria, which was highlighted in Additional File 1: Figure S2; Tables S2-S3). Based on these criteria, we selected bc 2.0 for all downstream analyses in the manuscript (e.g., genome distributions, promoter accessibility differences, GWAS). We believe this may have gotten lost with all of the excessive detail provided. The only analysis where we used a different stringency threshold was calling footprints and the reason for this is described in the response below. The evaluations of different cut-offs, e.g., details on peak metrics (#, sizes, % genomic coverage), are still presented now in the Methods and Supplemental data.*

Line 174: This bar plot is not useful as a metaprofile would be to visualize the relative location of peaks to genes.

*Response: We present meta-profiles in Figure 2a and Supplemental Figure S5 showing chromatin accessibility with respect to genes. In our example in Figure 1, we also wanted to highlight the frequency of accessible regions found in introns compared to exons, etc. These profiles change when looking at accessible regions that are specific to tassel and ear in Supplemental Figure S4, and we’ve found the bar plot an intuitive way to visualize this.*

Line 182: 'intergenic' a confusing term here as all things in this plot are intergenic. Perhaps 'genomic distribution' or something else would be better. It took me a bit to understand this plot.

*Response: Thank you for the feedback here. We realize that this figure was not intuitive as previously presented. For Figure 1d we changed the y-axis to ‘distance to nearest PCG (kb)’ Beneath ear and tassel we included ‘intergenic HS) and changed ‘intergenic’ to ‘genomic midpoints. The figure legend was updated to better reflect this as well.*

Line 193: What are you doing with non-expressed genes? Are they in the low expression group or are they excluded?

*Response: They are in the low expression group. This is now indicated on line 180.*

Figure 2: The colors used here are difficult to separate. I would encourage the authors to use easily distinguished colors for the panels a and b.

*Response: Thank you for this suggestion. The colors have been changed for better resolution.*

Lines 208-214: This is a really interesting and important question. How well does the variation in accessibility associate with dynamic gene expression. I had a really tough time understanding the authors take-home point about this. To me it looks like the correlation is quite poor. Only a subset of regions with alter accessibility show altered expression and the directionality is not well correlated.

*Response: Overall, MNase hypersensitivity across promoters positively correlated with gene expression level, as shown in Figure 2a and Supplemental Figure S7.
Our analyses presented in Figures 2b-e were not performed to address this question further, but rather to identify specific regulatory regions that may control differences between tassel and ear morphology. This differential DNS analysis used only 500 bp of promoter sequence and we know that maize promoters extend further, so it would be less accurate to assess the correlation here. Instead, we identified particular regulatory regions in the proximal promoter for which accessibility was highly correlated, either positively or negatively, with gene expression differences between tassel and ear. This allowed us to break the groups down into potential activators or repressors of gene expression changes between tassel and ear primordia. These organs are almost identical developmentally, so we expect (and know from some examples) that regulation of gene expression is largely fine-tuning of shared pathways. The underlying regulatory elements (and the variation within) are important tools for engineering optimal inflorescence morphologies. This is the reason for the analyses around these candidate genes and gene sets.

To make this clearer overall to readers, we renamed this section “Differential HS in gene promoters reveals regulatory DNA associated with organ-specific expression”, which is more accurate and hopefully better conveys the significance of this section. We also reworded a few sentences in this section to clarify this in lines 185-190: “Genes that control meristem determinacy are largely expressed in both tassel and ear, but it is the precise regulation of these genes that fine-tune developmental processes that lead to subtle morphological differences. By defining differential promoter accessibility and/or usage coupled to gene expression changes, we hope to resolve tissue-specific regulatory differences that underlie variation in branching, or meristem determinacy, between tassel and ear.”*

Lines 215-229: This is not well justified. What are the authors trying to address here? They are focused on the subset of genes with altered accessibility and expression. Why are these more meaningful than if they looked at all genes with differential expression? As expected they find some TFs with known functions. The detailed analysis of the TCP genes really doesn’t seem to have a true take-home. Instead this paragraph seems to provide a random set of observations that re not really testing specific concepts.

*Response: In response to the first part of the question, please refer to the response above (regarding lines 208-214 and the next response below).
We do not agree with the reviewer’s assessment of this section and hope that with the changes made in response to the previous comment, the significance becomes clearer. As discussed in the major comment #3 above, we know little about the transcriptional regulators that modulate specific developmental processes in maize. There are over 2k TFs in the maize genome and we only have experimental evidence on what a very small fraction of them do. Context-specific interrogation of a specific developmental process through integration of relevant multi-omics data can shed light on function for potentially important regulators of unknown function. Our discovery here of 5 TCP family TFs (for 4 of them there is nothing known in the literature about their function) that all showed marked increases in expression and promoter accessibility in ear compared to tassel primordia was exciting since it provides biological insight into their individual functions in playing a role in repressing branching - this is at a time right when the primary branches are forming on the tassel and in ear they are repressed. While this makes sense for well-known tb1, the other 4 uncharacterized TCPs are all Class I TCPs, which have not previously been implicated in this process. Furthermore, in the next section of the Results, we found some of their accessible promoters were enriched for known TCP binding sites, suggesting higher orders of their regulation. We added in lines 214-215: “All five TCPs showed strikingly higher expression in ears and notable increases in promoter accessibility” to better highlight this finding.

In order to streamline the manuscript and highlight the most relevant discussions of biology, we removed some of the discussion on tassel-specific TF enrichment from the end of the Results section in question and several lines of extraneous details on TCPs from the Discussion.*

Lines 230-247: Again, what is the goal here? Why are these genes better than simply looking at differentially expressed genes?

*Response: Please see response to lines 208-214. As described above, our main interest was to define tassel- or ear-specific regulatory sequences, which can be used to help construct gene regulatory networks underlying meristem determinacy pathways, investigate for natural variation, or target for genome editing. The genes that are referred to in the manuscript as differentially regulated between tassel and ear provide a high-confidence set of loci, including both the gene and the proximal regulatory sequences that likely harbor binding sites of TFs that regulate it.*

These discussions of GO terms don’t seem to actually provide any insights.

*Response: We respectfully disagree here and believe that functional enrichment of genes with similar expression profiles in this context can help delineate key processes underlying differences in tassel and ear development at this critical stage of developmental patterning. Biological processes related to genes positively regulated in ear tend to be associated with organogenesis and determinacy (as expected) but also certain hormone and nutrient pathways. Those positively regulated in tassel were enriched for different processes, providing insight into distinct pathways underlying morphological and metabolic differences between the two structures.
We did remove another section with discussion of GO terms, which was also suggested by the reviewer (comment below).*

Lines 253-255: This is a really nice result and highlights the value of these HS sites in finding potential TF binding sites.

*Response: We appreciate the positive comment from the reviewer. We were also pleased to see this result and feel confident about these data in predicting genome-wide TF binding.*

Lines 258-260: I am not convinced this observation is useful. Why is it important to note a slight difference in binding site size? Is this statistically significant?

*Response: This result shows that in places where we have experimental evidence of two TFs binding in the same location, there is a higher degree of accessibility. Lines 494-495 read: “.. indicating that the degree of accessibility scaled with number of binding factors”. This is expected if the degree and area of accessibility are correlated with increased binding of TFs, in this case combinatorial binding. It is simply a qualitative observation, which provides additional support for the MNase data overlapping experimentally-validated TF occupancy.*

Lines 260-264: GO terms provide no value here.

*Response: Here, we agree with the reviewer that this particular GO enrichment analysis does not provide much valuable information aside from a small number of people who study KN1 and FEA4 TFs. This is now removed from the text.*

Lines 273-288: This is an interesting analysis. What proportion of the FEA4 accessible regions contain this motif? If you divide the genes near these peaks into those with and without ABI3 motif do these two groups of genes show similar or distinct expression patterns in your samples?

*Response: 70% of the FEA4 accessible regions contain the ABI3 motif. With respect to the FEA4 consensus, 39% contain ABI3 motif within 100 bp (28% within 50 bp). This is now noted in line 261: “This motif was present within 70% of the FEA4 binding site regions”.
We thought the latter suggestion was a great one – we looked at gene expression based on RNA-seq data across a series of early tassel and ear developmental stages. We didn’t observe anything to note in gene expression patterns among the entire set of genes, but perhaps subsets (e.g., those associated with a particular pathway) may be more informative. The GO enrichment analysis we presented (Figure 3e) for the genes proximal to the FEA4-ABI3 cis module showed overrepresentation of ABA-related GO terms, for example, in comparison to random FEA4 motifs of the same size.*

Lines 293-296: Why use a higher (bc4) cut-off here? What about KN1 binding sites?

*Response: For the footprint analysis we used bc 4 to increase the signal to noise ratio. This was performed on the genomic fragments identified genome-wide using bc 2. We used fragment centers to a higher bc value together to focus the peaks for motif discovery, which we believe should increase the likelihood of detecting the occupied motifs and decrease the likelihood of detecting motifs covered by broader and weaker peaks. The bc 4 threshold restricts analysis to regions with the highest depth of read coverage. Without a ground truth cistrome occupancy dataset to train against, it is difficult to quantify and optimize for signal to noise ratio.

Indeed, we detected the KN1 binding site enriched in proximity to FEA4 binding sites. This is shown in Supplemental Figure S10. It is now clarified in the text on line 259.*

Lines 297-307: I don’t understand why groups of genes with positive and negative associations are grouped together here. It seems you’d have more power to focus solely on the groups of genes for which there is a positive or negative association separately.

*Response: Our interest in doing the analysis this way was to identify tassel- or ear-specific regulation, either through gene activation or repression. We have shown in past publications that TFs that play important developmental roles can act as either activators or repressors of gene expression (Eveland, et al., Genome Res, 2014). Here we are focused on identifying differential regulation in tassel or ear regardless of mode of action. Certainly, genes can be grouped together in various ways for this type of analysis depending on the question of interest. We reworded the sentence on lines 280-281 to clarify this: “We next performed motif enrichment analysis within the footprints to predict candidate TF binding that drives suites of differentially regulated genes between tassel and ear primordia”.*

Lines 313 and on: Why are these enhancers? Aren’t they as likely to be distal repressors as enhancers?

*Response: This is a good point and we completely agree. This was our oversight. We have revised the wording of enhancers to “distal regulators” or similar in places where they can either be considered enhancers or repressors.*

Lines 318-320: The evidence for lncRNA overlap with distal regulators in plants is relatively sparse. Many distal enhancers have little or no lncRNA detected.

*Response: We agree with the reviewer that there are few functionally validated examples of this in plants and by no means do we want to suggest that this is a common phenomenon. However, there are some very compelling examples, including APOLO in Arabidopsis (Ariel et al., Mol. Cell, 2014). Here, our goal is to provide the processed data sets, their integration, and some discussion of biological context as a resource for mining targets that can be followed up on experimentally by researchers in the community. This is further expanded on in the response to the next comment.*

Lines 322-380: This analysis [analysis of inflorescence-expressed lncRNAs] really does not seem to fit well within this paper. It is not clear why they did not use previous lncRNAs that included these tissues and many others. Similarly, the analysis of chromatin near lncRNAs has been documented in several plants including maize. This whole section really does not associate these lncRNAs to the accessible regions the authors are studying or compare lncRNAs from accessible regions to those from inaccessible regions.

*Response: We appreciate the reviewer’s comments, which suggest that the significance of this dataset was perhaps not clearly defined or well-integrated. The major goals of this manuscript are i) to provide a high-resolution, integrated genomics resource for optimal mining of the regulatory space during a specific, critical time in the development of maize tassel and ear primordia, and ii) to highlight observed differences between them. In addition to TF-DNA interactions, non-coding RNAs contribute significantly to this regulatory landscape. Therefore, our analyses of early inflorescence lncRNAs are aimed to provide an annotated resource to the community, and their surrounding chromatin profiles provide support for their regulation, which can also be further used to identify testable hypotheses about their regulation.

It is known that lncRNAs are largely not evolutionarily conserved and show high spatiotemporal specificity. It is this specificity that allows them to fine-tune gene expression in various ways. We are well aware of earlier studies looking at lncRNAs in maize (and cite them within the manuscript) and although a few used RNAseq datasets from immature inflorescences, the stages are not overlapping. There is a large developmental timeline from SAM transition to pollination, and the datasets we used here are specific for early developmental transitions in mostly meristematic tissue. We show in Figure 5 and in Supplemental Figure S14 that across a developmental series of staged tiny tassel and ear primordia, there are highly specific lncRNAs that are spatiotemporally expressed even in our data set. We further show correlations in gene expression between these lncRNAs and protein coding genes across a developmental trajectory. These data integrated with TF-DNA associations predicted from the genome-wide accessibility profiles will help researchers in mapping out regulatory modules controlling these agronomically important developmental processes.
In order to convey this more clearly to readers, we altered the text in lines 305-309 to now read: “Since lncRNAs are highly context-specific, we cataloged the spatiotemporal expression of lncRNAs in maize inflorescence primordia to i) investigate their association with intergenic HS chromatin signatures and ii) to extend our regulatory maps for tassel and ear development.”*

Lines 383-410: This is not a novel finding. The Stam and Jiang groups nicely demonstrated that many accessible regions are located within annotated transposons. The results in this work do not provide additional information beyond these earlier works and those are not cited here.

*Response: Our novel finding here was that the increased accessibility in intergenic regions we observed in tassel compared to ear (Figure 1d) was associated with retrotransposons. This observation is supported by recent findings that transposon activation in the male germ line occurs much earlier in maize inflorescence development compared to in Arabidopsis (Warman et al., PLOS Gen, 2020; Slotkin et al., Cell, 2009). We appreciate this comment, however, since it suggests (as in the response to Reviewer 2) that the discussion of tassel vs. ear transposon accessibility should come earlier in the manuscript where the discrepancy between tassel and ear intergenic regions is introduced. It also suggests that the transposon section needs to be highly condensed to only highlight this novel finding. We are well aware of the previous work in this area (citations at line 168), so we agree that this should be condensed. The entire “Increased intergenic chromatin accessibility in immature tassels associates with transposable elements” section now appears in the first part of the Results in lines 164-173. The data are still included as a Supplemental Figure now S6.*

Lines 412-455: This was an interesting analysis. There are many issues with GWAS implementations and the idea here to focus on accessible regions to improve detection was interesting. Once again, the authors switch to a different cut-off (bc2).

*Response: We appreciate the positive response from the reviewer. We believe that more ways that we can use biological insight to guide GWAS analyses, the more we can improve detection of causal loci.
The use of the bc 2 cut-off throughout the manuscript has been clarified (see response above).*

Lines 439-455: The authors really do not provide evidence for improvement of outcomes using their approach. As expected, some of the best candidates overlap [between the two approaches]. The authors find some cases in which the same gene was identified as significant due to different SNPs. How often does this occur? The authors describe an example of this but don’t specify the frequency that this happens and can’t resolve which SNP is more likely causative. Finally, the authors mention several candidates found using the MNase SNPs but not the genome-wide examples. This is cherry picking of some examples. This really isn’t a good quantitative estimate of the value of these approaches.

*Response: We appreciate the reviewer’s comments here but we respectfully disagree that this is cherry picking. All of the genes associated with GWAS SNPs from both analyses and their overlaps are provided in Additional File 15: Dataset S14. There were 17 genes associated with different SNPs from the 2 analyses. GWAS is an inherently conservative analysis, where typically only the largest-effect signals are declared to be statistically significant. Thus, there is a critical need to reduce the conservativeness of GWAS by looking at biologically relevant subsets the genome so that other signals of smaller effect (albeit still biologically meaningful) can be identified. We argue that this paper itself provides a biologically meaningful justification in focusing on MNase-only regions, particularly since candidate genes identified by using only MNase regions make sense biologically. Of course, these need to be functionally validated to really know for sure.

We previously discussed the ‘can’t resolve the causative SNP’ question in the Discussion. Since the analysis was performed using the NAM population, we expect that the haplotype blocks will limit our ability to resolve causal SNPs, regardless of sub-setting the functional genome. We hypothesized that this may work best in a large association panel.
To address the comment on how to test for improvement of outcomes using MNase-only SNPs, we conducted an additional analysis in a small maize diversity panel where public phenotype data was available. In brief, this additional analysis suggested that tassel MNase regions tended to harbor associations with lower P-values than equally-sized random subsets of markers. This supports the idea that using only MNase SNPs does indeed seem to prioritize functional SNPs, lowers the significance threshold required to declare a marker significant, and thus could reduce the computational load for genomic selection models in a large association panel. This analysis now appears at the end of the results on lines 412-424 and in Supplemental Figure S16. The discussion section on this was condensed into the results and lines 545-550 in the Discussion.*

Reviewer 2:
The study by Parvathaneni, Bertolini et al using differential nuclease sensitivity mapping to reveal the regulatory landscape of developing maize inflorescences. Chromatin accessibility was identified in a manner that enabled footprint resolution providing data necessary for the construction of some key transcriptional modules that are important to the development of maize ear/tassels. The regions of differential nuclease sensitivity were highly concordant with in vivo ChIPseq data of key transcription factors (KN1/FEA4) supporting the high quality nature of the accessible chromatin regions identified. Additionally, a class of inflorescence enriched lncRNAs were also captured in this study. They were annotated and possible regulatory roles were explored. Lastly, focusing on traits associated with the tissues examined the intersection of trait associated SNPs from previously published GWAS and the newly identified accessible regions were evaluated. This analysis revealed a significant enrichment of trait associated SNPs in differential nuclease sensitivity sites.

Overall, the use of differential nuclease sensitivity mapping to identify the cis-regulatory landscape of the development of maize inflorescence tissues represents a valuable contribution to the field. This study is timely and it incorporates tissue specific DNA footprint analysis that enables the exploration of transcriptional factor regulatory pathways important to development of these tissues. The comments below are intended to improve this already strong manuscript.

*Response: We appreciate the reviewer’s enthusiasm for the work! In response to the comment on “previously published GWAS data” - we want to emphasize that the GWAS analyses were novel to this work. We used publicly available phenotype data for NAM and performed a stepwise model selection procedure for TBN tassel branch number (TBN) and ear row number (ERN) with the HapMapv3 SNPs. There have not been published studies for TBN or ERN using this marker set, therefore, we provide a new resource of associated SNPs for these inflorescence traits. Lines 376-378 state “To test this, we first performed a GWAS analysis for two inflorescence traits, TBN and ERN, using publicly available phenotype data from the NAM population and HapMap v3 SNP markers”.*

1 – Footprint analysis using DNase I and/or Tn5 has revealed that some sites are sensitive to the biases associated with the enzyme being used. One way to identify these sites is to use a genomic DNA control and to perform footprint analysis on the regions found from using chromatin as the input material. Is it a concern when using MNase and if so, what is the most appropriate control to manage enzymatic biases in nuclease activity that plagues DNase-seq and ATAC-seq?

*Response: This is an interesting question and while easily asked, there are a lot of assumptions that may not be valid when trying to answer it as posed. The sequence bias of MNase has been defined on naked DNA in solution, not cross-linked chromatin in nuclei. It is not clear how biochemically relevant that bias is to the ability of MNase to cut DNA beside occupied particles in chromatin. In a recent and thorough Genome Biology paper from the Jiang lab (MSU; Zhao et al., 2020), MNase sensitivity in cross-linked chromatin was compared to DNase I HS-seq and ATAC-seq datasets and shown to be comparable, with even higher spatial resolution, in identifying regulatory regions. We note that these findings were published (as is the case for nearly all MNase-seq data sets) without any controls to manage enzymatic bias. It may be that a proper control would involve somehow assembling nuclear chromatin in which nucleosome and non-nucleosomal particles are present at normal genomic abundance but at randomly distributed locations prior to nuclease treatment.
Indirect evidence that MNase is not plagued by false positives associated with enzymatic bias may be seen by the observations that it has been used to detect tissue-specific changes in chromatin structure (Turpin et al., 2018, DIB, 4 tissues), where the underlying sequence composition is NOT changing, and tissue-specific detection by MNase of differential TF occupancy (Vera et al., Plant Cell, 2014; Rodgers-Melnick et al., PNAS, 2016; Zhao et al., Genome Biol., 2020) in maize and treatment-specific changes in chromatin occupancy associated with light-regulated transcriptional changes in Arabidopsis (Pass et al., PLOS Genet, 2017).

Consequently, we are not certain that any such bias plagues our data or those of others and do not believe that slight preferences of MNase for different sequence composition on naked DNA can be meaningfully used as a control.*


2 – It is stated that there is a high rate of false negatives from peak identification of nuclease sensitive sites. What is the evidence that adding more reads solves this solution, as replicates were combined for most analyses? Can individual replicates be used for analysis and figure creation (such as figures 2b and 5c) instead of combining them (with the exception of footprinting where deep data is required)?

*Response: The statement on false negatives was perhaps framed poorly to suggest that we know the rate is high. We see potential false negatives in certain cases when we look gene by gene, but combining replicates allowed us to simplify things, including visualization of results, since the two replicates were well-correlated. And even though the tissue is largely homogeneous since it is meristematic, there are various cell types present, which can dampen the signal. In addition, each replicate is composed of ~170 pooled individuals, and so is an average across a large population anyway. We wanted to achieve the most depth possible when looking for TF binding sites. This is something we now reworded for clarity now found in the Methods section lines 647-648: “Manual inspection in a genome browser revealed several cases where MNase HS was visible in the two biological replicates, but only called as a peak in one likely due to coverage threshold”. And lines 649-651: “To enhance depth of coverage for resolution of dynamic accessibility and footprints of bound TFs, we also combined reads from the two replicates and iSeg was used to call MNase HS on the combined datasets as well as for individual replicates.”

We incorporated suggestions from the reviewer to use individual replicates for the analyses presented in Figure 2b, which is now updated. We really appreciate this suggestion as we believe it adds value to the manuscript, allowing readers to assess the consistency between replicates across promoters of differentially expressed genes for tassel and ear. In 5c, however, tassel and ear showed the same pattern anyway (as did replicates) and visualization got too busy to be meaningful so we kept the combined distributions in here. We also provide coordinates of high confidence accessible regions that are called in both replicates as a .bed file that can be downloaded and displayed on a browser to (Additional File 4: Dataset 3). Line 645 reads: “..DNS peaks overlapped between biological replicates and high-confidence HS regions were called if their midpoints were within 300 bp of each other.”*

3 – Line 183. What is the biological rationale for why ear and tassel peak distance would be so different? Is this somehow related to the technical quality of the data instead of the biology?

*Response: We are glad that the reviewer brought this up since we were worried about a disconnect on this point with the previous version of the manuscript. Our hypothesis for why there were more intergenic peaks found in tassel was that they were largely associated with retrotransposons. This is supported by data in this manuscript and also in a recent paper by Warman et al., PLOS Gen., 2020, that showed early activation of transposons in tassel primordia. In the previous version of the manuscript, we did not mention this hypothesis until way later into the results where we discussed transposons.
Because of this confusion and in response to reviewer #1, we condensed the transposon section substantially and moved it up front to support this hypothesis when showing the distribution data between tassel and ear. This is now described in lines 164-173.*

4 – I’m confused why mid points were used for identification of DNA footprints. Why can’t the first base be used to indicate regions that are sensitive to nuclease activity.

*Response: Footprints assume occupancy for which the underlying cis-elements tend to be centrally located. Furthermore, plotting fragment centers removes some peak artifacts that result from overlapping ends of two fragment populations whose midpoints are close together. This is particularly true for genome-wide, biochemically-defined fragments from multicellular tissues. The first base does not as accurately capture the location of the underlying motif as well as the fragment center. Focusing on fragment centers should reduce the detection of "adjacent" or "nearby" motif detection, as is commonly found in promoters and transcriptional CRE hubs.*

5 – Please report how many intergenic lncRNAs were removed from the final list due to protein-coding capacity. This is especially important given the known issues with the annotation of the maize genome.

*Response: Supplemental Figure S12 reports the detailed number of transcripts that were filtered out during the lncRNA annotation pipeline. 15,606 potential noncoding transcripts were discarded due to protein coding capacity. This is now also stated in the methods section line 748-749.*

6 – Line 346. Around 30-40% of the enhancers identified from reference 16 are enriched for unannotated genes and lncRNAs. Therefore, they are not classic enhancers.

*Response: We appreciate the reviewer’s comment here and understand that this certainly could be the case. The list of putative maize enhancers published in Genome Biology by Oka et al. 2016 was based on DNase I hypersensitivity assay, methylation profiles, and various histone marks indicative of enhancers. There have not been any updates or corrections to this paper and we use this peer-reviewed resource as a comparative dataset to our own. As more related studies emerge comparing various tissues, etc., as well as experimental validation, we anticipate a clearer view of enhancer loci in the maize genome.*

Most of the opening of the results section could be moved to the methods section.

*Response: We completely agree with this and appreciate the suggestion to make the manuscript more readable. Much of this has been condensed and moved to the methods now lines 632-653.*

Change “linc” to “lnc”.

*Response: Thank you for this suggestion – we agree that it reads better with this standardized, even though ‘lincRNA’ is technically a correct abbreviation for long intergenic noncoding RNA. We did update according to the reviewer’s suggestion throughout the manuscript.*


Reviewer 3:
The manuscript "The regulatory landscape of early maize inflorescence development" was a pleasure to read. Very important work, outstanding representation in the figures and an amazing wealth of data to dive in and continue the quest to understand and manipulate the switch between the female and male flowers in maize.
This paper is massive also in its current form and I needed several breaks to fully digest the content while reviewing. I could not find any concerns with the work or methods.
My only minor concern is the length and the possibility that valuable information might not be fully utilized by our research community simply because it is "hidden". The presented Discussion is a review paper in itself, and the Results sections could be split in two strong research papers. Just saying...

*Response: We really appreciate the enthusiastic comments from the reviewer regarding the work and its biological impact! We do absolutely agree that there was too much detail in certain parts of the original manuscript (as was suggested by all 3 reviewers), including excessive detail on methods and biological cut-offs in the beginning of the results, as well as a long winding discussion that went into detail in too many areas of the biology. The updated manuscript has been condensed and streamlined for improved readability. Specifically, we moved a large portion of the discussion on methodology from the Results to the Methods and condensed it. We also removed the section on the potential biological significance of the FEA4 and ABI3 combinatorial motifs from the Discussion. We took out the section associating MNase HS with transposons and moved a highly condensed version of the most important take-home to the beginning of the Results section. We also condensed the discussion on GWAS in the Discussion, moving some of it in a condensed form to the Results where we provide supporting information on the GWAS experiment. Throughout the manuscript we also removed several blocks of text that go into excessive detail about biological insight (that can otherwise be gleaned from going through the figures), but kept the things we thought most relevant to the biology.
We also tried to highlight the valuable take-homes by framing our biological questions more clearly in each of the sections.*

**Second round of review**

**Reviewer 1**

The authors have done a number of revisions to address my previous comments as well as those from the other reviewers.  I remain convinced that this could be trimmed further and that some of the analyses could be reduced further or removed to improve the work.  However, I will leave that to the discretion of the authors and editor.  I will focus on a couple of issues that I think are important to resolve for clarity of presentation or accuracy.  Most important is the last issue mentioned below.  The GWAS section is not effectively testing the stated hypothesis or providing the interpretation the authors are making.

Line 150-152: A set of random regions matching the sizes of the MNase HS sites should be used and these numbers should be provided in figure 1C to see how the distributions compared to a random set of regions.  

Lines 167-169: I am not sure I understand this test and I don’t think this is true as currently written.  The text suggests that “Based on TE annotations in the maize genome we found enrichment of MNase HS associated with TEs…”. This seemingly suggests that since 85% of the maize genome is TE derived that >85% of MNase HS regions are in TEs.  I don’t think that is true and I don’t think that was shown in previous studies either.  Please clarify exactly what is being reported here.  I do believe the authors assertion that tassel has more TE accessibility than ear tissue but I am not sure how they are testing for enrichment relative to random regions.

Lines 197-198 add figure 2C: The colors in this figure are difficult to distinguish.  I appreciate the layout of the figure but I struggle to actually quantify what the authors are saying in the text.  What number of genes are correlated and what number are anti-correlated?  This can be specified in the text.

Lines 235-246:  This is a really nice bit of evidence to support the use of the LCS regions.  I got a little confused reading the text and looking at the figures (3a and 3b).  All of these analyses are only looking at the LCS reads (less than 130bp) right?  In the text the authors often use the phrase MNase HS but this was used in multiple ways and I wanted to confirm this is only LCS regions.  I am curious about the point being shown in figure 3b.  The authors are arguing that the MNase HS signatures are broader than those both by multiple TFs.  Is there a concern than in many cases in which there are multiple TFs bound you would generate fragments >130bp and miss some of these signals?

Lines 272-288: This is a really nice analysis.  It suggests focusing on the middle of these regions can be really useful.  I really like this but just had one question.  What proportion of these LCS peaks might be due to combinatorial binding of multiple TFs?  If multiple TFs are bound would that suggest that maybe the mid-point is not ideal because the two TFs would not bind exactly in the middle?  I am just curious given the argument from the previous section showing many FEA4 peaks also have KN1 or maybe an ABI3-like binding site.

Lines 348-366: I understand that the authors wanted to uncover lncRNAs specifically in the tissues of interest.  However, they are quite underpowered to do a co-expression analysis by restricting to this small subset of tissues.  If they really want to do this test they should use a much broader set of tissues and stages to really confirm the co-expression of lncRNAs and PCGs.

Lines 370-424:  this analysis remains problematic.  The authors want to test the hypothesis that “using only SNPs associated with regions of chromatin accessibility in a GWAS model would help resolve novel SNP-trait associations, including smaller effect loci that may drive more subtle phenotypic variation”.  This is an important hypothesis and worth testing.  However, the current implementation does not do this effectively.  They are basically testing two very different size SNP sets.  This should be done in a different way.  The full Hapmap v3 SNP set is worth doing.  However, when doing the accessible chromatin SNPs they need to include a control of a set of random regions that cover similar space as the accessible regions (or to subsample a set of SNPs that is the same size as that present in the accessible regions).  Without this the authors are not comparing equivalent things.  Furthermore, the authors really do not provide evidence that their accessible GWAS hits are any better than the full SNP set GWAS hits.  There are a subset of hits that identify genes in both approaches and the authors now quantify theser.  However, there are also many examples of GWAS hits only in the full set or only in the HS set.  If the HS set was doing a better job and picking up hits with smaller effects then it should capture everything in the full model as well as additional hits. I am fine with the discussion that maybe researchers should prioritize SNP associations within HS regions but am not convinced that doing this actually captures the phenotypic variation better.

**Reviewer 2**

I really like the revised version of this manuscript. It has been streamlined, which i think better highlights the novel results presented here.

**Authors Response**

**Point-by-point responses to the reviewers’ comments:**

*We appreciate the additional comments and suggestions from the reviewer since they allowed us to further clarify points in the manuscript, including rewording text and adding specifics to methods. Regarding the GWAS, we had previously performed the analysis that the reviewer is asking for in a 282-line maize association panel. In this revised manuscript, we further refined our analysis in the 282 panel to assess the performance of a GWAS model using only MNase HS-associated SNPs compared to genome-wide subsets. Since we used a stepwise model selection procedure to call trait-associated SNPs in NAM, some of the expectations from a single-step GWAS model (e.g., same SNPs should be present in both models) do not hold true and this may have caused confusion (described in response to reviewer comments below). We have addressed all of the reviewer’s comments/questions below and noted where specific revisions have been made to the manuscript text, methods, and supporting documents.*

******************************************************************************
Line 150-152: A set of random regions matching the sizes of the MNase HS sites should be used and these numbers should be provided in figure 1C to see how the distributions compared to a random set of regions.

*Response: We performed this analysis as per the reviewer’s request. As anticipated, the majority of random genomic fragments (MNase HS region-sized) fall overwhelmingly in the intergenic space, since this makes up most of the maize genome. We provide below the relative proportions of random MNase region-sized fragments falling into the genomic features examined for the reviewer to check. Comparing these to the numbers we show in Figure 1c, there is clearly MNase HS enrichment in regions flanking genes and UTRs compared to random genomic regions. We have to argue that we do not see the utility in having these numbers in the figure. In Figure 1c, total number of MNase HS sites mapping to a particular feature were normalized based on the total length in bp of that feature in the maize genome. This is a typical presentation of the data in published studies of accessible chromatin in various species. We believe that Figure 1c provides straightforward results that show the distribution of MNase HS across genomic features in the maize genome and which features are preferentially MNase HS.

normalized in 10kb
ear; tassel
exon: 0.651824267; 0.771464406
intron: 1.195676639; 1.401812929
5-UTR: 1.391083224; 1.471725729
3-UTR: 1.164187821; 1.371521507
2kb-up: 1.184771781; 1.361906465
promoter: 1.17685372; 1.100227325
1kb-down: 1.17672601; 1.626778371
intergenic: 2.355971124; 2.747983476*

***********************************************************************************

Lines 167-169: I am not sure I understand this test and I don’t think this is true as currently written. The text suggests that “Based on TE annotations in the maize genome we found enrichment of MNase HS associated with TEs…”. This seemingly suggests that since 85% of the maize genome is TE derived that >85% of MNase HS regions are in TEs. I don’t think that is true and I don’t think that was shown in previous studies either. Please clarify exactly what is being reported here. I do believe the authors assertion that tassel has more TE accessibility than ear tissue but I am not sure how they are testing for enrichment relative to random regions.

*Response: We appreciate the reviewer’s attention to detail here and in helping us clarify this. The main issue here is that we accidentally left this detail out of the methods. We tested for enrichment of MNase HS within TEs relative to random intergenic regions as background by running a permutation test (n = 1,000).
We updated this in the Methods, lines 679-681, which now reads: “The enrichment analysis was conducted using the Bioconductor package regioneR [106] based on permutation tests (n = 1000). A randomization strategy to compare intergenic genomic regions was performed using the function circularRandomizeRegions.”
To further clarify this in the text, the sentence in line 168-169 now reads “Based on TE annotations in the maize genome [41], we found enrichment of MNase HS associated with TEs relative to randomized intergenic regions…”.*

**********************************************************************************
Lines 197-198 and figure 2C: The colors in this figure are difficult to distinguish. I appreciate the layout of the figure but I struggle to actually quantify what the authors are saying in the text. What number of genes are correlated and what number are anti-correlated? This can be specified in the text.

*Response: This information is included in Additional File 8: Dataset S7. In fact, we found a mistake in the previous version of the manuscript - these numbers are available in Additional file 8: Dataset S7 and NOT in Dataset S8 as was previously written. This has now been updated on line 198.

Of the 794 genes that showed expression differences between tissues AND substantial differences in accessibility (based on criteria in Methods) within 500 bp upstream of their TSS, 494 were up-regulated in ear. This is consistent with an induction of a determinacy program in ear. When comparing numbers of genes that showed direct correlation of expression with promoter accessibility between the two organs, we see approximately half are associated with positive regulation (49.2%) and the other half negative regulation (50.8%). This makes sense given that the few TFs we have looked at using ChIP-seq in these tissues can function as both activators and repressors in a spatiotemporal manner to drive developmental programs.
However, as we mentioned previously, it is misleading to consider these numbers as a complete representation in trying to correlate accessibility with gene expression between tissues since we know that expression of a given gene is typically controlled much more broadly than in the 500 bp upstream of the TSS. The correlation between promoter accessibility and degree of gene expression is clearly shown for both tassel and ear in Figure 2a and Supplemental Figure S7, respectively. We want to be careful to not overinterpret our results in Figure 2c as a global indicator for the relation of accessible chromatin to gene expression, but rather use them to identify regulatory elements that we can begin to interrogate for their roles in regulation of gene expression between tassel and ear, and particularly distinguish between activator and repressor binding. We expect that gene expression differences between these tissue types are linked largely to fine-tuning of developmental processes, which can be complex in their regulation.

We have now provided this information at the end of the second sentence in lines 198-201: “Approximately 41% (794) showed substantial expression differences between tassel and ear, including direct correlations with chromatin accessibility as well as anti-correlated trends (Figure 2c). The latter could be indicative of repressor binding and this was observed for approximately half of the genes.”*

********************************************************************************
Lines 235-246: This is a really nice bit of evidence to support the use of the LCS regions. I got a little confused reading the text and looking at the figures (3a and 3b). All of these analyses are only looking at the LCS reads (less than 130bp) right? In the text the authors often use the phrase MNase HS but this was used in multiple ways and I wanted to confirm this is only LCS regions.

*Response: Yes, this analysis was based on LCS regions. This was stated in line 238 of the text (right at the beginning of the section) as well as in the figure legend. To further clarify and avoid confusion, we updated Figure 3a to now specify “No overlap with MNase HS (LCS)” and the y axis in Figure 3b now reads “Read density (LCS)". We also made the same updates to Supplemental Figure S9.*

********************************************************************************
I am curious about the point being shown in figure 3b. The authors are arguing that the MNase HS signatures are broader than those bound by multiple TFs. Is there a concern than in many cases in which there are multiple TFs bound you would generate fragments >130bp and miss some of these signals?

*Response: In Figure 3b, the distribution of read densities covers a broader area on average in the promoters of genes bound by both FEA4 and KN1 TFs when compared to genes classified as bound by one (KN1) or the other (FEA4). This observation is interesting given that we have so few examples genome-wide ChIPseq profiles in maize. The fact that we have shown that KN1 and FEA4 occupy many of the same promoters in these tissues allows us to infer that there may be more regulatory activity at these co-bound regions, e.g., combinatorial regulation, which is supported by the MNase-seq data. It is indeed possible that if truly co-occupied sites close together protected a DNA fragment larger than our 130 bp cutoff, it would be excluded such that yes, we may miss them. However, because of our lack of resolution with the ChIP-seq data (e.g., coming from pools of cell types), we do not know how these two TFs interact on the DNA, and it likely differs in various spatiotemporal contexts. The term "co-bound" here, as also defined and used in (Pautler, Eveland et al., 2015), refers to overlapping peaks when projected onto a singular genome assembly representation. They may, but formally may not co-occur on the same exact DNA fragment (e.g., they may compete for the DNA site depending on the cell type within the meristem). And if so, it does not necessarily mean that the two proteins are so close as to occlude cutting between them, if indeed they co-occupy a region.*

*******************************************************************************
Lines 272-288: This is a really nice analysis. It suggests focusing on the middle of these regions can be really useful. I really like this but just had one question. What proportion of these LCS peaks might be due to combinatorial binding of multiple TFs? If multiple TFs are bound would that suggest that maybe the mid-point is not ideal because the two TFs would not bind exactly in the middle? I am just curious given the argument from the previous section showing many FEA4 peaks also have KN1 or maybe an ABI3-like binding site.

*Response: These are really great questions that we too are curious about, but do not have the specificity (e.g., deep cell-specific profiles) nor enough knowledge of examples of cis-elements with specific co-bound occupancy and varying spacings to answer with certainty. TF binding is likely fluid and dynamic such that at any given time (i.e., at the point of formaldehyde crosslinking) a large number of specific but different binding configurations may be captured. The previously noted complication also comes into play here, namely that "overlapping” ChIP-seq peaks typically span large regions due to DNA shear size techniques, where the bound protein could be anywhere along those molecules, often hundreds of bp long. Further development of small particle footprinting within accessible chromatin regions in maize, such as work underway by HW Bass et al. (unpublished, manuscript in preparation), may help address this curiosity. However, a slightly broader peak that covers 2 motifs will not prevent the motifs from being detected as "under the peaks", or if concerned, it is easy to expand the peak to 50bp regions, but that increases the risk of detecting nearby, but not occupied sites.

For the sake of speculation, the fragment pattern for two adjacent TFs may appear as three closely-spaced peaks that may be merged into one. There could be two from closely spaced fragment center peaks for each of the two TFs, and a third, possibly less frequent central peak from individual molecules that were cut once to the left of the first TF and once to the right of the second TF, but not in between. Given that the LCS are a subset of the light digests, we likely do not have the depth to reliably know for sure, and prefer not to over-speculate, tempting as it is.*

********************************************************************************
Lines 348-366: I understand that the authors wanted to uncover lncRNAs specifically in the tissues of interest. However, they are quite underpowered to do a co-expression analysis by restricting to this small subset of tissues. If they really want to do this test they should use a much broader set of tissues and stages to really confirm the co-expression of lncRNAs and PCGs.

*Response: We agree with the reviewer here that a co-expression network analysis that included a larger breadth of relevant tissues and time points may provide more resolution for which lncRNAs may interact with which PCGs in a larger context. That being said, deciding what goes into a co-expression analysis is always subject to availability of samples, nature of the question, etc. Here, we are particularly interested in the correlation in expression pattern of lncRNAs and proximally positioned PCGs across very specific stages of inflorescence development - specificity that may not be resolved when looking at a larger range of tissues. Of course, the lncRNAs annotations are now available through this manuscript for researchers to explore their co-expression profiles within the context of various network options.
To help clarify this so as to not suggest we are performing a co-expression network analysis, we adjusted line 351 to read “we analyzed correlation of their expression profiles ..” in place of “we analyzed their co-expression profiles”.*

********************************************************************************
Most important is the last issue mentioned below. The GWAS section is not effectively testing the stated hypothesis or providing the interpretation the authors are making.

Lines 370-424: this analysis remains problematic. The authors want to test the hypothesis that “using only SNPs associated with regions of chromatin accessibility in a GWAS model would help resolve novel SNP-trait associations, including smaller effect loci that may drive more subtle phenotypic variation”.
This is an important hypothesis and worth testing. However, the current implementation does not do this effectively.

*Response: We realize that some of our wording in this section suggested the rigorous testing of a hypothesis. While we did test the hypothesis that MNase guided GWAS can improve resolution of trait-associated SNPs with lower p-values, and also showed evidence for MNase markers explaining more of the phenotypic variance than random markers in a 282 line maize association panel, we cannot say which of the two GWAS models tested in NAM is “better” without experimental validation beyond the scope of this manuscript. This is described in more detail below.
To clarify this better in the text, we changed line 376 to “.. using only those SNPs associated with regions of chromatin accessibility in a GWAS model could potentially help resolve novel SNP-trait associations ..”. And in line 378, we changed “To test this” to “To investigate this”.*

********************************************************************************
They are basically testing two very different size SNP sets. This should be done in a different way. The full Hapmap v3 SNP set is worth doing. However, when doing the accessible chromatin SNPs they need to include a control of a set of random regions that cover similar space as the accessible regions (or to subsample a set of SNPs that is the same size as that present in the accessible regions). Without this the authors are not comparing equivalent things.

*Response: In our previous revision of the manuscript we actually performed the experiment that the reviewer is describing in a small association panel, the Goodman-Buckler association panel (n = 282). We showed that by performing GWAS using a Benjamini-Hochberg correction with 71,024 SNPs that fell within MNase HS regions compared to the same number of random markers genome-wide, we achieved more significant SNPs with substantially higher FDR adjusted p-values. This was described in the text and presented in Supplemental Figure S16.

In this new revision, we further addressed this in the Goodman-Buckler association panel by generating 1,000 random subsets of SNPs of equivalent size to the MNase-accessible SNP subset (n = 71,024) and calculated FDR-adjusted p-values separately for each subset using the Benjamini-Hochberg procedure. While we cannot know for certain whether one subset is “better” than another without knowledge of the underlying QTN, we used the smallest (most significant) 1% of FDR-adjusted p-values for each subset as a proxy for the “quality” of the SNP subset, and found that the MNase-accessible SNP subset contained far more highly significant p-values than any of the 1,000 random subsets of equivalent size. This value is represented by a statistic we denote as lambda (λ), which is a measure of the decrease in FDR adjusted p-values for the 99th percentile of the reduced marker set compared to that using genome-wide FDR adjusted p-values on the same marker set. The λ for the tassel MNase SNP subset was four standard deviations higher that the λ range for random subsets.

This equation is provided now in Supplemental Figure S16, along with the updated analysis, and in the Methods section lines 821-831. This is now described in the text lines 414-431.

This result suggests that, based on our observations in the 282-line Goodman-Buckler maize association panel (where TBN phenotype data is available), using markers in MNase HS regions achieved lower adjusted p-values for more SNPs associated with TBN.*

*******************************************************************************
Furthermore, the authors really do not provide evidence that their accessible GWAS hits are
any better than the full SNP set GWAS hits.

*Response: We cannot determine which model in NAM is “better” without knowledge of the underlying QTN. We do know that the MNase-only SNP model hit genes that we know are involved in inflorescence architecture that were not uncovered using the genome-wide analysis nor in other QTL studies for inflorescence architecture traits in maize prior.*

********************************************************************************
There are a subset of hits that identify genes in both approaches and the authors now quantify these. However, there are also many examples of GWAS hits only in the full set or only in the HS set.
If the HS set was doing a better job and picking up hits with smaller effects then it should capture everything in the full model as well as additional hits.

*Response: This (same SNPs picked up in both models) would indeed be true for a single-step GWAS analysis, such as the one that we performed with the Goodman-Buckler association panel, but not for the stepwise model selection procedure that was performed in the NAM population of RILs (n= 5,000). Using this procedure, markers are included and excluded based on their significance. Therefore, the significance of a QTL is dependent on the previous markers in the model. In the stepwise NAM analysis, if the first step of the “All SNPs” model selects a SNP that is not present in the “MNase SNPs” subset, then all subsequent steps of the model will likely differ between the “All SNPs” and “MNase SNPs” analyses. If you have a reduced marker set there is no guarantee you will get the same QTL. Furthermore, regardless of GWAS method used, we also do not necessarily expect that every QTN will fall in a HS region, including large-effect loci, which could also result in different sets of candidate genes between GWAS models.
This is now emphasized in the text at lines 546-548: “Since a stepwise model selection procedure was used, we would not expect the genome-wide model to include all SNPs from the MNase-guided model”.

A stepwise analysis allows one to build a model that contains multiple SNPs/QTL, and you can see how much total variation they explain. By including the effects of big QTL in the model, you can sometimes then detect additional QTL with smaller effects, that would not otherwise be detected with a single marker analysis. A statistical model with multiple SNPs is a better representation of reality where multiple genes contribute to phenotypic variation. Using a stepwise selection procedure for GWAS in NAM is considered the gold standard for maize and is extremely computationally intensive. Therefore, our intention in this manuscript is to provide high-quality genetics data for inflorescence architecture to the maize community based on two different GWAS models in NAM. We hope that by following up on the SNP associations presented in one or both models, new insights into the control of inflorescence architecture in maize will be accelerated. Our analysis of MNase SNPs vs genome-wide subsets in the 282 association panel provide the statistical evidence that MNase-guided GWAS performs “better” in terms of providing more significant markers with lower p-values associated with a trait of interest. Compared to randomly selected SNP subsets of identical size, our results suggest that MNase regions represent a functional subset of the genome and that this smaller subset can potentially be substituted for the genome-wide marker set in marker-assisted selection protocols.

As mentioned in the Results lines 414-415, we expect that identifying causal SNPs by selecting for only MNase will be less clear in the NAM population due to linkage disequilibrium, and more relevant in an association panel. Our use of the Goodman-Buckler panel here is a proxy for this.*

********************************************************************************
I am fine with the discussion that maybe researchers should prioritize SNP associations within HS regions but am not convinced that doing this actually captures the phenotypic variation better.

*Response: We will not know if we are capturing the phenotypic variation better until we experimentally validate genes associated with the SNPs identified in our models. Our intent is to use these results (and provide them to the community) for hypothesis generation. Follow-up studies on SNP associations unique to the MNase-accessible SNP subset, such as those in bif1, rel2, and sbp3, are beyond the scope of this manuscript. However, we note that the developmental windows during which TBN and ERN are determined in maize have been exceptionally well-characterized, and we believe that SNPs responsible for regulatory effects on phenotype would be captured here.
Our analyses on the 282 panel showed that MNase accessible regions are potentially useful for identifying more SNPs associated with TBN that would normally have fallen below the significance threshold in a genome-wide analysis.*

**Third round of review**

**Reviewer 1**

I appreciate the authors providing additional clarification and revisions.  While some of the specific choices about presentation of the results may not be the path I would take, the authors have explained the basis of their choices.  The revisions have significantly improved the clarity and accuracy of the GWAS results.  The revised version more clearly delineates the results from NAM and the 282 panel.  More importantly, the language in the current manuscript has removed some of the adjectives (ie better) that were not well supported initially and provides a more accurate description of the results.  I do not have further issues that need to be addressed - thank you for your revisions.
